# IL-4 Haplotype -590T, -34T and Intron-3 VNTR R2 Is Associated with Reduced Malaria Risk among Ancestral Indian Tribal Populations

**DOI:** 10.1371/journal.pone.0048136

**Published:** 2012-10-24

**Authors:** Aditya Nath Jha, Vipin Kumar Singh, Namrata Kumari, Ashish Singh, Justin Antony, Hoang van Tong, Sakshi Singh, Sudhanshu S. Pati, Pradeep K. Patra, Rajender Singh, Nguyen L. Toan, Le H. Song, Amal Assaf, Iara J. T. Messias–Reason, Thirumalaisamy P. Velavan, Lalji Singh, Kumarasamy Thangaraj

**Affiliations:** 1 Centre for Cellular and Molecular Biology, CSIR, Hyderabad, India; 2 Institute of Tropical Medicine, University of Tübingen, Tübingen, Germany; 3 Ispat General Hospital, Rourkela, Orissa, India; 4 Pt. Jawaharlal Nehru Memorial Medical College, Raipur, India; 5 Central Drug Research Institute, CSIR, Lucknow, India; 6 Department of Pathophysiology, Vietnam Military Medical University, Hanoi, Vietnam; 7 Tran Hung Dao Hospital, Hanoi, Vietnam; 8 Department of Laboratory Medicine, University of Damascus, Damascus, Syria; 9 Laboratório de Imunopatologia Molecular-Hospital de Clínicas, Federal University of Paraná, Curitiba, Brazil; 10 Banaras Hindu University, Varanasi, India; University of Hyderabad, India

## Abstract

**Background:**

Interleukin 4 (IL-4) is an anti-inflammatory cytokine, which regulates balance between T_H_1 and T_H_2 immune response, immunoglobulin class switching and humoral immunity. Polymorphisms in this gene have been reported to affect the risk of infectious and autoimmune diseases.

**Methods:**

We have analyzed three regulatory *IL-4* polymorphisms; -590C>T, -34C>T and 70 bp intron-3 VNTR, in 4216 individuals; including: (1) 430 ethnically matched case-control groups (173 severe malaria, 101 mild malaria and 156 asymptomatic); (2) 3452 individuals from 76 linguistically and geographically distinct endogamous populations of India, and (3) 334 individuals with different ancestry from outside India (84 Brazilian, 104 Syrian, and 146 Vietnamese).

**Results:**

The ***-***590T, ***-***34T and intron-3 VNTR R2 alleles were found to be associated with reduced malaria risk (P<0.001 for ***-***590C>T and ***-***34C>T, and P = 0.003 for VNTR). These three alleles were in strong LD (r^2^>0.75) and the TTR2 (***-***590T, ***-***34T and intron-3 VNTR R2) haplotype appeared to be a susceptibility factor for malaria (P = 0.009, OR = 0.552, 95% CI = 0.356 –0.854). Allele and genotype frequencies differ significantly between caste, nomadic, tribe and ancestral tribal populations (ATP). The distribution of protective haplotype TTR2 was found to be significant (χ^2^_3_ = 182.95, p-value <0.001), which is highest in ATP (40.5%); intermediate in tribes (33%); and lowest in caste (17.8%) and nomadic (21.6%).

**Conclusions:**

Our study suggests that the *IL-4* polymorphisms regulate host susceptibility to malaria and disease progression. TTR2 haplotype, which gives protection against malaria, is high among ATPs. Since they inhabited in isolation and mainly practice hunter-gatherer lifestyles and exposed to various parasites, *IL-4* TTR2 haplotype might be under positive selection.

## Introduction

*Plasmodium falciparum* malaria is one of the major causes of morbidity and mortality in tropical and sub-tropical areas [1]. Despite the significant advances in disease control, *Plasmodium falciparum* malaria accounts for 1–3 million deaths annually [2]. The variation in the severity of *Plasmodium falciparum* infections include different phenotypes such as hyper or asymptomatic parasitaemia, mild malaria, severe malaria and cerebral malaria [3], [4] and the host genetic architecture contribute to these malarial phenotypes [5]. Increasing epidemiological and experimental evidences suggest that the host genetic variations play an essential role to thwart actively or passively the parasite invasion. [4]. The fundamental attribute of the innate immune system is to recognize pathogen and react swiftly to contain the early infection while signaling to specific adaptive immune response. Studies have investigated role of innate immune genes such as Toll-like receptors (TLR2, 4, 9), chemokines and cytokines role in *Plasmodium falciparum* malaria [6]. In addition, plethora of studies have documented that genetic heterogeneity in many immune genes is associated with malaria susceptibility [3].

Malarial infection is characterized by pro-inflammatory responses during early stages of infection followed by anti-inflammatory responses during disease progression [7]. The human Interleukin 4 (*IL-4*) located in the chromosome 5 (5q31-33), is an anti-inflammatory cytokine produced by CD4+ Th2 cells, basophils and mast cells. IL-4 regulates variety of cell types [8] and play an essential role in differentiation of Th2 effector cells, suppression of Th1 signaling, promoting humoral immunity and Ig class switching and a dominant role in immunopathology [9], [10], [11]. Studies have revealed that INF-γ levels were significantly elevated during early stages of malaria, whereas the IL-4 levels were elevated during intermediate and late stages indicating a switch towards Th2 response [12]. A significant inverse correlation between IL-4 to INF-γ ratio and peripheral parasitaemia in malaria patients has been documented [13].

Human *IL-4* gene promoter contains six conserved binding sites of NFAT (nuclear factor of activated T-cells), a transcription factor; along with activator protein 1 (AP1) regulates *IL-4* transcription [14]. Studies have shown that the -590C/T transition creates a seventh NFAT binding site and synergistically up regulates *IL-4* transcription rate up to 3 fold [15]. Studies have documented that elevated antibody IgG and IgE levels against malaria antigens, parasitaemia and malaria susceptibility has been associated with *IL-4* -590T allele in several African populations [8], [16], [17], [18], [19]. The -590T allele is believed to be under positive selection in various populations and indicates local adaptation to diverse pathogenic challenges [20]. Further, -34T promoter polymorphism association with elevated total serum IgE levels has been demonstrated [8]. Also the presence of H3K27Ac mark was observed near active regulatory element of intron-3 VNTR region in different cell lines (www.ucsc.edu). Studies have established that IL-4 as a key regulator in malaria and three regulatory *IL-4* polymorphisms (-590C/T, -34C/T and in intron-3 VNTR) have been shown to regulate serum IL-4 levels, IgG, IgE, disease progression and survival [16], [17], [18], [19], [21], [22], [23], [24], [25]. Also the three regulatory polymorphisms in the *IL-4* loci were associated with end stage renal disease, multiple sclerosis, autoimmune Grave’s disease, chronic polyarthritis, rheumatoid arthritis, asthma, rhinitis and atopic dermatitis [21], [26], [27], [28], [29].

Although several studies have documented on functional significance of *IL-4* polymorphisms in different ethnicities, to the best of our knowledge no studies have investigated the contribution of *IL-4* variants in Indian population. Indian populations remain isolated from rest of the world for thousands of years and are unique in their origin and have accumulated unique set of mutations and the variants influencing disease susceptibility among Indian populations remain different compared to other ethnicities [30]–[31]. In this study, we aim to investigate the contribution of three functional *IL-4* polymorphisms rs2243250 (-590 C>T, promoter), rs2070874 (-34 C>T, 5′UTR) and rs79071878 (intron-3, 70 bp VNTR) with *P. falciparum* malaria infection in well-defined malaria cases and in ethnically matched controls. Since the entire Indian subcontinent represents a malaria endemic region, we extended our investigation of the three functional *IL-4* polymorphisms to different linguistically and geographically isolated Indian populations and compared the observed differences to that of different ethnicities representing world populations.

**Table 1:** Characteristics of studied subjects segregated according to clinical classification.

	Sample Size	Mean Age (year)± SD	Male: Female
Asymptomatic	156	29.87±19.57	92∶64
Mild Malaria	101	30.24±15.71	67∶34
Severe Malaria	173	26.11±12.38	103∶70

## Materials and Methods

### Study Subjects

A total of 4216 individuals were investigated for *IL-4* gene polymorphisms (-590C/T, -34C/T and in intron-3 VNTR). Of which 173 individuals were clinically characterized with *Plasmodium falciparum* severe malaria and 101 with mild malaria and 156 with asymptomatic individuals from *P. falciparum* endemic states, Orissa and Chhattisgarh (Table 1). We also utilized 3452 individuals from 76 distinct populations representing caste (n = 1568), nomadic (n = 114), tribes (n = 517) and ancestral tribal populations [(ATP) (n = 1253)] of India. Also three world populations representing tropical regions such as Brazil (n = 84), Syria (n = 104) and Vietnam (n = 146) were included in this study (Table 2).

**Table 2:** Characteristics of studied subjects of various world populations.

Ethnicity	Population	No. of individuals
India	Caste	1568
	Nomadic	114
	Tribe	517
	Ancestral tribes	1253
Brazil	Brazil	84
Syria	Syria	104
Vietnam	Vietnam	146

### Sampling

All individuals representing the malaria cohort were clinically classified. Classification of malaria was carried out on WHO guidelines; severe malaria (n = 173) is defined as severe anemia (hemoglobin <50 g/l) and/or hyper-parasitemia (>250,000 parasites/µl, corresponding to >10% infected erythrocytes), a Blantyre coma score ≤2 and other facultative signs of severe malaria such as cerebral malaria, convulsions, hypoglycemia, and respiratory distress. All individuals were hospitalized for treatment. Mild malaria (n = 101) is defined as parasitemia 1000–50,000/µl on admission, no schizontaemia, circulating leukocytes containing malarial pigment <50/µl, not homozygous for hemoglobin S, hemoglobin >80 g/l, platelets >50/nl, leukocytes <12/nl, lactate <3 mmol/l, and blood glucose >50 mg/dl. Asymptomatic individuals (n = 156) were characterized as individuals harboring parasites without clinical signs during sample collection. Intra-venous blood sample (∼5 ml) was collected from each individual admitted at Ispat General Hospital, Rourkela, India; and Pt. Jawaharlal Nehru Memorial Medical College, Raipur, India.

**Figure 1:**
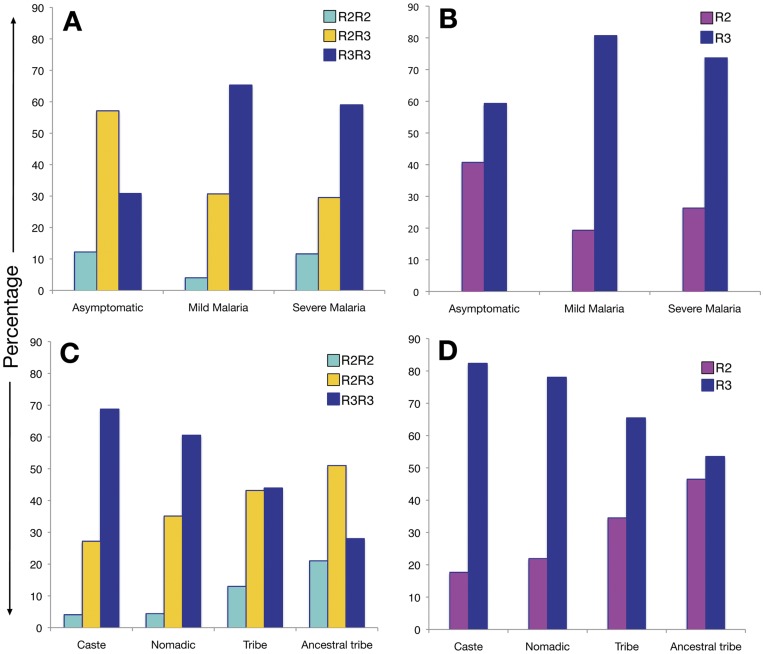
Distribution of *IL-4* intron-3 VNTR polymorphism. A and B: genotype and allelic distribution between malaria case control groups, respectively; C and D: genotype and allelic distribution among caste, nomadic, tribe and ancestral tribe, respectively.

**Figure 2:**
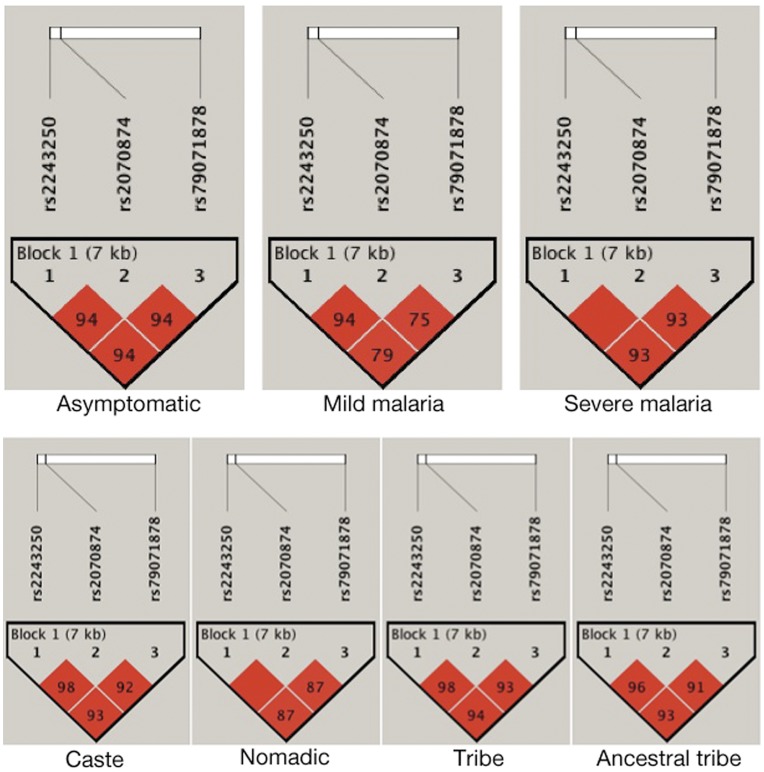
Linkage Disequilibrium (LD) of studied *IL-4* loci (-590 C/T, -34 C/T and intron-3 VNTR) in malaria case control and Indian population groups. The three studied SNPs are in strong LD (r^2^>0.75, malaria case control groups; r^2^>0.87, population groups).

### Ethical Committee Approval

An informed written consent was obtained from every individual. The study was approved by the Institutional Ethical Committee (IEC) of Centre for Cellular and Molecular Biology, Hyderabad, India; Ispat General Hospital, Rourkela, Orissa, India; and Pt. Jawaharlal Nehru Memorial Medical College, Raipur, Chhattisgarh, India.

### Genotyping of SNP and VNTR Variants

Genomic DNA was extracted from whole blood using the protocol as previously described [32]. To re-sequence 800 bp of *IL-4* promoter region, we utilized the reference sequence from the ENSEMBL (ID: ENSG00000113520; www.ensembl.org). The sequence specific primer pairs were designed using the Primer-BLAST (http://www.ncbi.nlm.nih.gov/tools/primer-blast), MacVector (MacVector, Inc. USA) and the Amplify 3X (http://engels.genetics.wisc.edu/amplify) software platforms.

DNA was amplified using a primer pair spanning the promoter regions to detect polymorphisms at -590C/T and -34C/T variants. The primer pairs employed were IL-4_promo_F: 5′-TATGGACCTGCTGGGACCCAAACTA-3′, and IL-4_promo_R: 5′-CACCTTCTGCTCTGTGAGGCTGTTC-3′ (Eurofiins mwg operon). In brief: 5 ng of genomic DNA was amplified in a 10 µl reaction volume using Qiagen long-range PCR kit following manufacturers instructions (Qiagen, Germany) on a GeneAmp 9700 Thermal cycler (ABI, USA). Thermal cycling parameters for amplification were: initial denaturation at 94°C for 3 min, followed by 35 cycles of 30 sec at 93°C denaturation, 25 sec at 66°C annealing, 1 min 30 sec at 68°C extension, followed by a final extension of 5 min at 68°C. PCR products were cleaned up using Exo-SAP-IT (USB, Affymetrix, USA) and 1 µl of the purified product were directly used as templates for sequencing, using the BigDye terminator v. 3.1 cycle sequencing kit Applied Biosystems, USA) on an ABI 3730XL DNA sequencer, according to the manufacturer’s instructions. DNA polymorphisms were identified when assembled with the reference sequence using AutoAssembler software (Applied Biosystems).

The variable nucleotide repeat regions (VNTR) in the intron-3 were amplified using primer pairs IL-4_del_70_F: 5′-GCCTTTAGATTCCACCACGAGTATG-3′ and IL-4_del_70_R: 5′-GGTCATCTTTTCCTCCCCTGTATCTTA-3′. PCR products were size fractionated on 2% agarose gel to detect the repeat polymorphisms. PCR amplicons of 389 bp (two repeats each of 70 bp) were designated as R2 whereas amplicons of 459 bp (three repeats, each of 70 bp) as R3. A subset of samples were reconfirmed and validated for their R2 and R3 polymorphisms by direct sequencing.

**Table 3:** Distribution of IL4 genotypes and alleles in patients with malaria and in asymptomatic controls.

SNP ID and Polymorphism	Genotype (%)			Genotype Comparison		Allele (%)		Allele Comparison	
				?^2^_4_	P-value			?^2^_2_	P-value
**rs79071878, R2/R3**	R2R2	R2R3	R3R3			R2	R3		
Asymptomatic	19 (12.2)	89 (57.1)	48 (30.8)			127 (40.7)	185 (59.3)		
Mild Malaria	4 (4.0)	31 (30.7)	66 (65.3)			39 (19.3)	163 (80.7)		
Severe Malaria	20 (11.6)	51 (29.5)	102 (59.0)	42.2	<0.001	91 (26.3)	255 (73.7)	30.3	<0.001
**rs2070874, -34C/T**	TT	TC	CC			T	C		
Asymptomatic	15 (9.6)	83 (53.2)	58 (37.2)			113 (36.2)	199 (63.8)		
Mild Malaria	2 (2.0)	39 (38.6)	60 (59.4)			43 (21.2)	159 (78.7)		
Severe Malaria	19 (11.0)	54 (31.2)	100 (57.8)	25.3	<0.001	92 (26.6)	254 (73.4)	13.6	<0.001
**rs2243250, -590 C/T**	TT	TC	CC			T	C		
Asymptomatic	15 (9.6)	80 (51.3)	61 (39.1)			110 (35.3)	202 (64.7)		
Mild Malaria	4 (4.0)	37 (36.6)	60 (59.4)			45 (22.3)	157 (77.7)		
Severe Malaria	19 (11)	54 (31.2)	100 (57.8)	19.5	<0.001	92 (26.6)	254 (73.4)	11.3	0.003

### Statistical Analysis

The allele and genotype frequencies were analyzed by simple gene counting and expectation-maximum (EM) algorithm and the significance of deviations from Hardy-Weinberg equilibrium was tested using the random-permutation procedure as implemented in the Arlequin v.3.5.1.2 software (http://cmpg.unibe.ch/software/arlequin3/) [33]. Pairwise Fst values and co-ancestry coefficient were calculated using Arlequin using un-phased data Linkage disequilibrium (LD) analysis was performed using Haploview v4.2 software [34]. The allele and genotype distribution were calculated by chi square test in different sample sets using the SPSS (ver. 20). In all analysis, a two tailed p-value less than 0.05 were considered significant. Chi square contingency-table test results were interpreted by standardized residual method of post hoc analysis [35]. Probable effect of sex stratification and sample size were verified by bootstrap (10000 random sampling events) with bias-corrected and accelerated (BCa) method, using SPSS (ver. 20).

**Table 4:** Genotype comparison among various groups of malaria case control and population study in multiple stages.

Group 1 Vs. Group 2		R2R2, R2R3, R3R3		(R2R2+R2R3) Vs. R3R3	
		?^2^_2_	p-value	?^2^_1_	p-value
Malaria case-control groups					
Asymptomatic	Mild Malaria	30.2	<0.001	28.3	<0.001
Asymptomatic	Severe Malaria	28.9	<0.001	25.1	<0.001
Mild Malaria	Severe Malaria	4.6	0.097	1.1	0.293
Asymptomatic	Pooled Cases	38.0	<0.001	35.8	<0.001*
Population groups					
Caste	Nomadic	3.48	0.175	2.9	0.08
Caste	Tribe	118.9	<0.001	101.4	<0.001
Caste	ATP	482.5	<0.001	437.9	<0.001
Tribe	ATP	44.32	<0.001	40.0	<0.001

* OR = 3.566, 95% CI = 2.348–5.416;

## Results

### Role of IL-4 Variants in Malaria

The distributions of *IL-4* genotype and allele frequencies are summarized in Table 3 and Figure 1. Mainly two copies and three copies of 70 bp repeat (intron-3 VNTR) has been observed in humans and are designated as R2 and R3 respectively, whereas only a single copy of 70 bp repeat has been observed in other primates (Figure S1, www.ensembl.org). The genotype frequencies of intron-3 VNTR polymorphism differed significantly among ethnically matched asymptomatic controls, individuals with mild and severe malaria (χ^2^_4_ = 42.2; p<0.001). Promoter polymorphisms -590 C>T and -34 C>T also differed significantly among the studied malarial sub groups (χ^2^_4_ = 19.5; p<0.001 and χ^2^_4_ = 25.3; p<0.001). Similarly, allele distributions also differed significantly among these groups (intron-3 VNTR: χ^2^_2_ = 30.3; p<0.001, -590 C>T χ^2^_2_ = 13.6; p<0.001 and -34 C>T χ^2^_2_ = 11.3; p = 0.003). All three studied loci were in HW equilibrium and were in strong LD (r^2^>0.75) (Figure 2) with two major haplotypes, CCR3 and TTR2 identified (Table S2). Therefore, analysis has been performed only with VNTR polymorphism or the resulting haplotypes. Significant difference has been observed in the distribution of these haplotypes between cases and asymptomatic control (OR = 0.552, 95% CI = 0.356−0.854, p = 0.009) (Table S2) with TTR2 as protective haplotype.

**Figure 3:**
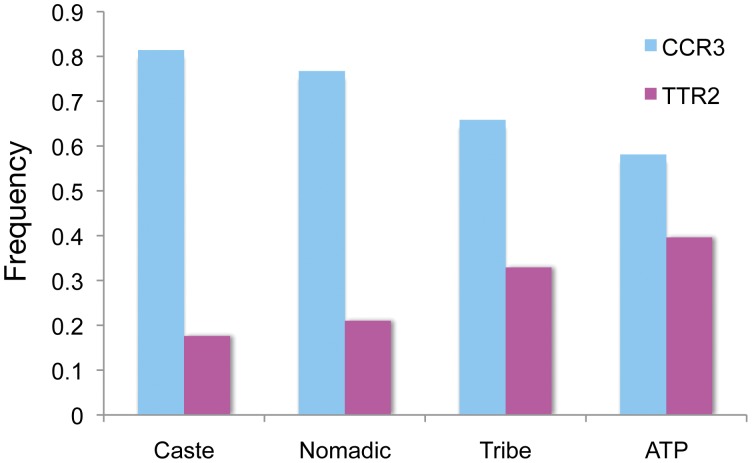
Haplotype distribution among various Indian social groups. The prevalence of protective haplotype TTR2 is significantly higher in ancestral tribal populations.

Post-hoc analysis of chi-square contingency-table test showed over representation of genotype R3R2 (57.1%; z score = 5.5) and under representation of R3R3 (30.8%; z score = −6.1) genotypes in asymptomatic control; while over representation of R3R3 [(mild, 65.3%; z score = 3.5), (severe, 59.0%; z score = 3.0)] and under representation of R2R3 [(mild, 30.7%; z score = −2.1), (severe, 29.5%; z score = −3.6)] and R2R2 [(mild, 4%; z score = −2.3) (severe, 59.0%; z score = 0.9)] in mild and severe malaria group and mainly contributes to the Chi-square [(Figure 1), (Table S1)]. Comparison of mild and severe malaria genotype distribution shows non-significant difference between these two groups χ^2^_2_ = 4.6; p = 0.097; while significant difference was observed between asymptomatic *vs*. mild (χ^2^_2_ = 30.2; p<0.001) and asymptomatic *vs.* severe malaria (χ^2^_2_ = 28.9; p<0.001) (Table 4). Since it has been reported that TT homozygous and CT heterozygous at position -590 and -34, R2R2 and R2R3 genotype leads to higher serum IL-4 level [22], we pooled R2R2 and R2R3 for further analysis. Again, comparison of R2R2+R2R3 *vs.* R3R3 between various groups show statistically significant difference, except between mild and severe malaria χ^2^_1_ = 1.1 p = 0.293 (Table 4). Therefore, we pooled mild and severe group together and compared with asymptomatic control, which came out to be highly significant [χ^2^_1_ = 35.8; OR = 3.566, 95% CI = 2.348–5.416 p<0.001] (Table 4).

As the proportion of male sample were higher in asymptomatic as well as in case groups, we further analyzed the distribution of R2/R3 in these groups for the possible effect of sex stratification. The frequency of protective allele R2 was found significantly lower in both the male and female groups of mild and severe malaria compared to asymptomatic control and rule out the possibility of sex stratification (Table S3).

### Association of Haplotype TTR2 with Ancestral and Tribal Indian Population

Among 3613 individuals from a total of 76 endogamous Indian ethnic populations investigated, we found striking pattern of genotype prevalence based on their social status and inhabitation. Most of the studied populations were in HW equilibrium (Table 5) and those, which were not in HW, were excluded for further analysis. Based on their social status, inhabitation, co-ancestry coefficient and pairwise F_st_ (Figure S2), we initially grouped these populations into three groups; namely, caste, nomadic and tribal populations. Caste is further grouped in to traditionally upper, middle and lower caste populations to see if there is any difference between these groups. Our study did not find any significant difference in genotype distribution among these groups (Figure S3). Further, tribal group has been divided into two groups the tribe (started agriculture recently) and the ancestral tribal population [(ATP), which includes all hunter-gatherer tribal populations]. The genotype distribution of these three loci (-590C/T, -34C/T and the intron-3 VNTR) differed significantly among the four classified sub groups (χ^2^_6_ = 501.1; p<0.001, χ^2^_6_ = 326.9; p<0.001 and χ^2^_6_ = 323.2; p<0.001) respectively (Table 6). In addition, allelic distributions differed also significantly (Table 6). We found these markers in strong LD having r^2^>0.87 (Figure 2) with significant difference in haplotype distribution among these four subgroups (χ^2^_3_ = 182.95, p-value <0.001) (Table S2). The protective haplotype TTR2 has been found to be highest in ATP (40.5%); intermediate in tribes (33%); and lowest in caste (17.8%) and nomadic (21.6%) (Figure 3).

**Table 5:** Social status, geographical origin, ethnicity, linguistic affiliation and HW equilibrium of Indian populations included in present study.

S. No.	Population	SocialDesignation	LanguageFamily	State	SampleSize	R2R2(%)	R2R3(%)	R3R3(%)	Obs.Het.	Exp.Het.	P-value
1	Kapu	Caste	Dravidian	Andhra Pradesh	57	2(3.51)	17(29.82)	38(66.67)	0.298	0.303	1
2	Reddy	Caste	Dravidian	Andhra Pradesh	56	1(1.79)	14(25)	41(73.21)	0.25	0.247	1
3	Vysya	Caste	Dravidian	Andhra Pradesh	48	0(0)	8(16.67)	40(83.33)	0.167	0.154	1
4	Yadav	Caste	Dravidian	Pudduchery	48	1(2.08)	7(14.58)	40(83.33)	0.146	0.171	0.3365
5	Achari	Caste	Dravidian	Tamil Nadu	71	3(4.23)	17(23.94)	51(71.83)	0.239	0.273	0.3714
6	Gugavellar	Caste	Dravidian	Tamil Nadu	68	1(1.47)	11(16.18)	56(82.35)	0.161	0.174	0.4696
7	Lingayat	Caste	Dravidian	Tamil Nadu	47	1(2.13)	12(25.53)	34(72.34)	0.255	0.256	1
8	Muppanar	Caste	Dravidian	Tamil Nadu	15	1(6.67)	4(26.67)	10(66.67)	0.267	0.331	0.4612
9	Muthaliar	Caste	Dravidian	Tamil Nadu	43	3(6.98)	11(25.58)	29(67.44)	0.256	0.319	0.0855
10	Pillai	Caste	Dravidian	Tamil Nadu	79	4(5.1)	17(21.52)	58(73.42)	0.215	0.266	0.2676
11	Sonakar	Caste	Indo-European	Chhattisgarh	41	0(0)	11(26.83)	30(73.17)	0.268	0.235	1
12	Lohana	Caste	Indo-European	Gujarat	46	1(2.17)	11(23.91)	34(73.91)	0.239	0.245	1
13	Tadavi	Caste	Indo-European	Gujarat	22	0(0)	8(36.36)	14(63.64)	0.363	0.301	0.3141
14	Bania	Caste	Indo-European	Hariyana	34	1(2.94)	10(29.41)	23(67.65)	0.294	0.295	1
15	Pandit	Caste	Indo-European	Hariyana	15	1(6.67)	3(20)	11(73.33)	0.2	0.287	0.3250
16	SC	Caste	Indo-European	Hariyana	40	2(5)	11(27.5)	27(67.5)	0.275	0.308	0.5995
17	Kamboj	Caste	Indo-European	Haryana	100	0(0)	30(30)	70(70)	0.3	0.256	0.1169
18	KashGujjar	Caste	Indo-European	Kashmir	44	3(6.81)	12(27.27)	29(65.91)	0.273	0.315	0.6439
19	KashMushlim	Caste	Indo-European	Kashmir	57	2(3.51)	21(36.84)	34(59.65)	0.368	0.345	1
20	KashPandit	Caste	Indo-European	Kashmir	47	2(4.26)	14(29.79)	31(65.96)	0.298	0.312	0.6604
21	Baghel	Caste	Indo-European	Madhya Pradesh	35	0(0)	13(37.14)	22(62.86)	0.371	0.307	0.5678
22	Silawat	Caste	Indo-European	Madhya Pradesh	90	6(6.67)	31(34.44)	53(58.89)	0.344	0.383	0.8623
23	Jatav	Caste	Indo-European	Rajasthan	36	3(8.33)	10(27.78)	23(63.89)	0.2778	0.351	0.3241
24	Kurmi	Caste	Indo-European	Rajasthan	56	3(5.36)	14(25)	39(69.64)	0.25	0.296	0.3502
25	Meena	Caste	Indo-European	Rajasthan	41	1(2.44)	9(21.95)	31(75.61)	0.219	0.235	0.5384
26	Brahmins	Caste	Indo-European	Uttar Pradesh	59	2(3.39)	14(23.73)	43(72.88)	0.237	0.261	0.6056
27	Kshatriya	Caste	Indo-European	Uttar Pradesh	40	1(2.5)	8(20)	31(77.5)	0.2	0.221	0.4732
28	Khatri	Caste	Indo-European	Punjab	30	2(6.67)	8(26.67)	20(66.67)	0.267	0.325	0.3060
29	Bisht	Caste	Indo-European	Uttarakhand	45	1(2.22)	9(20)	35(77.78)	0.2	0.217	0.5007
30	Rawat	Caste	Indo-European	Uttarakhand	87	10(11.49)	34(39.08)	43(49.43)	0.391	0.430	0.4571
31	Darj_caste	Caste	Indo-European	West Bengal	32	3(9.3)	14(43.75)	15(46.88)	0.437	0.448	0.6923
32	Thapa	Caste	Tibeto-Burman	Nepal	39	3(7.69)	13(33.33)	23(58.97)	0.333	0.373	0.6607
33	Banjara	Nomadic	Indo-European	Rajasthan	42	1(2.38)	14(33.33)	27(64.29)	0.333	0.312	1
34	NariKuruwar	Nomadic	Indo-European	Tamil Nadu	72	4(5.56)	26(36.11)	42(58.33)	0.361	0.363	1
35	BodoGadaba	ATP	Austro-Asiatic	Andhra Pradesh	39	4(10.26)	26(66.67)	9(23.08)	0.667	0.498	**0.0501**
36	Chenchu	ATP	Austro-Asiatic	Andhra Pradesh	45	7(15.56)	17(37.78)	21(46.67)	0.378	0.457	0.3213
37	Asur	ATP	Austro-Asiatic	Jharkhand	45	6(13.33)	23(51.11)	16(35.56)	0.511	0.481	0.7568
38	Bhumij	ATP	Austro-Asiatic	Jharkhand	91	14(15.38)	42(46.15)	35(38.46)	0.461	0.476	0.8257
39	Birhor	ATP	Austro-Asiatic	Jharkhand	59	10(16.95)	27(45.76)	22(37.29)	0.457	0.483	0.7860
40	Kharia	ATP	Austro-Asiatic	Jharkhand	41	4(9.76)	22(53.66)	15(36.59)	0.536	0.470	0.5009
41	Parharia	ATP	Austro-Asiatic	Jharkhand	19	7(36.84)	11(57.89)	1(5.26)	0.579	0.462	0.3436
42	Saharia	ATP	Austro-Asiatic	Madhya Pradesh	30	3(10)	17(56.67)	10(33.33)	0.567	0.481	0.4446
43	Bondo	ATP	Austro-Asiatic	Orissa	40	8(20)	24(60)	8(20)	0.6	0.506	0.3411
44	Didayi	ATP	Austro-Asiatic	Orissa	30	8(26.67)	11(36.67)	11(36.67)	0.366	0.503	0.1575
45	Andamani	ATP	Andamanese	Andaman	18	1(5.56)	9(50)	8(44.44)	0.5	0.436	1
46	KondaReddy	ATP	Dravidian	Andhra Pradesh	15	4(26.67)	7(46.67)	4(26.67)	0.466	0.517	1
47	KondaSavara	ATP	Dravidian	Andhra Pradesh	93	37(39.78)	52(55.91)	4(4.3)	0.559	0.439	0.0890
48	Porja	ATP	Dravidian	Andhra Pradesh	69	23(33.33)	32(46.38)	14(20.29)	0.463	0.495	0.6314
49	Kattunayakan	ATP	Dravidian	Kerala	47	4(8.51)	22(46.81)	21(44.68)	0.468	0.439	0.7444
50	Paniyas	ATP	Dravidian	Kerala	33	7(21.21)	20(60.61)	6(18.18)	0.606	0.507	0.3072
51	Kuruman	ATP	Dravidian	Kerela	25	7(28)	12(48)	6(24)	0.48	0.509	1
52	Kurumba	ATP	Dravidian	Tamil Nadu	43	3(6.98)	21(48.84)	19(44.19)	0.488	0.436	0.4959
53	Kathodi	ATP	Indo-European	Gujarat	10	2(20)	4(40)	4(40)	0.4	0.505	0.5729
54	Kotwali	ATP	Indo-European	Gujarat	100	10(10)	52(52)	38(38)	0.52	0.463	0.2806
55	Katkari	ATP	Indo-European	Maharashtra	16	6(37.5)	6(37.5)	4(25)	0.375	0.508	0.3476
56	Baiga	ATP	Indo-European	Madhya Pradesh	60	9(15)	40(66.67)	11(18.33)	0.666	0.503	**0.0188**
57	Buxa	ATP	Indo-European	Uttar Pradesh	46	13(28.26)	26(56.52)	7(15.22)	0.565	0.497	0.3806
58	Onge	ATP	Jarwa-Onge	Andaman_nikobar	11	3(27.27)	7(63.64)	1(9.09)	0.636	0.506	0.5532
59	Mizo	ATP	Tibeto-Burman	Mizoram	15	8(53.33)	6(40)	1(6.67)	0.4	0.404	1
60	AoNaga	ATP	Tibeto-Burman	Nagaland	34	17(50)	12(35.29)	5(14.71)	0.352	0.425	0.2575
61	Juang	ATP	Tibeto-Burman	Orissa	69	15(21.74)	35(50.72)	19(27.54)	0.507	0.502	1
62	Korku	Tribe	Austro-Asiatic	Madhya Pradesh	42	6(14.29)	19(45.24)	17(40.48)	0.452	0.471	1
63	Koia	Tribe	Dravidian	Andhra Pradesh	86	6(6.97)	40(46.51)	40(46.51)	0.465	0.422	0.6098
64	Kotiya	Tribe	Dravidian	Andhra Pradesh	23	7(30.43)	11(47.83)	5(21.74)	0.478	0.507	1
65	Oddari	Tribe	Dravidian	Andhra Pradesh	19	2(10.53)	6(31.58)	11(57.89)	0.316	0.398	0.5492
66	Halaki	Tribe	Dravidian	Karnataka	36	1(2.78)	14(38.89)	21(58.33)	0.389	0.350	0.6563
67	Malayan	Tribe	Dravidian	Kerala	41	5(12.2)	21(51.22)	15(36.59)	0.512	0.476	0.7435
68	Muthan	Tribe	Dravidian	Kerala	61	7(11.47)	24(39.34)	30(49.18)	0.393	0.429	0.2782
69	Kolcha	Tribe	Indo-European	Gujarat	59	2(3.39)	30(50.85)	27(45.76)	0.508	0.413	0.1119
70	MahadeoKoli	Tribe	Indo-European	Maharashtra	51	8(15.69)	23(45.1)	20(39.22)	0.451	0.476	0.7695
71	Tharu	Tribe	Indo-European	Uttarakhand	48	11(22.92)	20(41.67)	17(35.42)	0.417	0.497	0.3758
72	Nyshi	Tribe	Tibeto-Burman	Arunachal Pradesh	10	8(80)	2(20)	0(0)	0.2	0.189	1
73	Darj_tribe	Tribe	Tibeto-Burman	West Bengal	11	3(27.27)	5(45.45)	3(27.27)	0.455	0.523	1
74	Malaikuruwar	ATP	Dravidian	Tamil Nadu	62	4(6.45)	5(8.06)	53(85.48)	0.081	0.189	**0.0006**
75	Toda	ATP	Dravidian	Tamil Nadu	48	3(6.25)	3(6.25)	42(87.50)	0.062	0.171	**0.0014**
76	Ulladan	Tribe	Dravidian	Kerala	30	1(3.3)	8(26.66)	21(70.00)	0.266	0.278	0.1613

R2: Two Repeat of IL-4 intro-3 VNTR, R3: Three repeat, ATP: Ancestral Tribal Population, Obs.Het : Observed heterozygocity, Exp.Het : Expected heterozygocity, P-value in bold shows departure from HW equilibrium at 0.05 level.

**Table 6:** Genotype and allele distribution of IL-4 intron-3 VNTR polymorphism, -34CT and -590CT in various groups of Indian population.

	Genotype (%)			Genotypic Comparison		Allele (%)			
				?^2^_6_	p-value			?^2^_3_	p-value
rs79071878, R2/R3	R2R2	R2R3	R3R3			R2	R3		
Caste	64 (4.1)	426 (27.2)	1078 (68.8)			554 (17.7)	2582 (82.3)		
Nomadic	5 (4.4)	40 (35.1)	69 (60.5)			50 (21.9)	178 (78.1)		
Tribe	67 (13.0)	223 (43.1)	227 (43.9)			357 (34.5)	677 (65.5)		
ATP	240 (21.0)	583 (51.0)	320 (28.0)	501.1	<0.001	1063 (46.5)	1223 (53.5)	537.6	<0.001
rs2070874, -34 C/T	TT	TC	CC			T	C		
Caste	67 (4.4)	443 (28.2)	1058 (67.4)			577 (18.4)	2559 (81.6)		
Nomadic	6 (5.3)	39 (34.2)	69 (60.5)			51 (22.4)	177 (77.6)		
Tribe	63 (12.2)	239 (46.2)	215 (41.6)			365 (35.3)	669 (64.7)		
ATP	205 (17.9)	519 (45.4)	419 (36.7)	323.2	<0.001	929 (40.6)	1357 (59.4)	347.9	<0.001
rs2243250, -590 C/T	TT	TC	CC			T	C		
Caste	71 (4.5)	439 (28.0)	1058 (67.5)			581 (18.5)	2555 (81.5)		
Nomadic	6 (5.3)	39 (34.2)	69 (60.5)			51 (22.4)	177 (77.6)		
Tribe	63 (12.2)	236 (45.6)	218 (42.2)			362 (35.0)	672 (65.0)		
ATP	212 (18.5)	514 (45.0)	417 (36.5)	326.9	<0.001	938 (41.0)	1348 (59.0)	351.4	<0.001

ATP: ancestral tribal population.

The genotype proportions between caste and nomadic did not differ significantly χ^2^_2_ = 3.48; p = 0.175, however, it differ significantly between caste *vs*. tribe; caste *vs*. ATP; and tribe *vs*. ATP with χ^2^_2_ = 118.9; p<0.001, χ^2^_2_ = 482.5; p<0.001 and χ^2^_2_ = 44.32; p<0.001, respectively (Table 4). We have also pooled R2R2 and R2R3 genotype together in agreement with higher IL-4 serum level [27] and compared with R3R3 genotype and found significant difference in genotype distribution between caste *vs*. tribe; caste *vs*. ATP; and tribe *vs*. ATP pairs with χ^2^_1_ = 101.4; p<0.001, χ^2^_1_ = 437.9; p<0.001 and χ^2^_1_ = 40.0; p<0.001, respectively (Table 4). Post-hoc analysis of chi-square contingency-table test result show over representation of genotype R3R3 (68.8%; z score = 19.6) and under representation of genotype R2R3 (27.2%; z score = −12.2) and R2R2 (4.1%; z score = −12.3) in caste while ancestral population ATP showed over representation of R2R3 (51.0%; z score = 11.1) and R2R2 (21.0%; z score = 12.9) and under representation of R3R3 (28.0%; z score = −18.9) genotype. Tribal group showed intermediate of caste and ATP [(R2R2, 13%; z score = 1.3), (R2R3, 43.1%; z score = 2.6), (R2R2, 43.9%; z score = −3.4)] (Figure 1) (Table S1).

**Figure 4:**
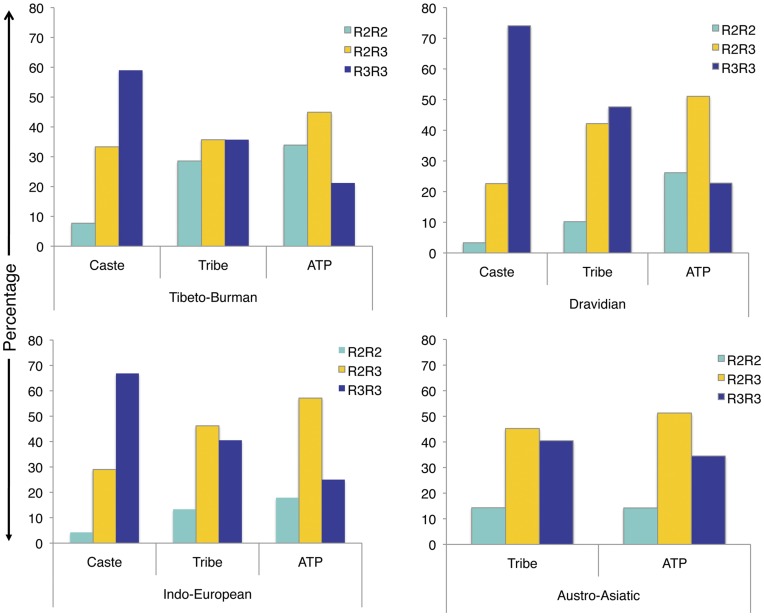
Genotype distribution of *IL-4* intron-3 VNTR polymorphism in four linguistic families of Indian populations.

Since there are four major linguistic families in India, we classified the samples in to four groups, namely; Indo-European (IE), Dravidian (DV), Austro-Asiatic (AA) and Tibeto-Burman (TB). Each linguistic family has been further classified in to caste, tribe and ATP. Interestingly, the difference in genotype distribution among caste, tribe and ATP follow the same pattern as of TTR2 haplotype distribution in ATP (highest), tribe and caste (lowest) populations (Figure 4).

### Geographical Distribution of IL-4 Variants

Comparison of all three studied variants to other world populations (Syria, Brazilian and Vietnamese) revealed diverse geographical patterns. We retrieved earlier reported data and compared with Indian population. We found Vietnamese has very high R2R2 (65.7%) genotype frequency, similar to other Southeast Asian countries. However, R2R2 genotype frequency in Syrian and Brazilian were only 4.8% and 6%, respectively (Figure S4).

## Discussion

We have screened the regulatory polymorphisms of *IL-4* promoter region (rs2243250, -590CT and rs2070874, -34 CT) and intron-3 repeat region (rs79071878, 70 bp VNTR) to investigate their possible role in survival against disease and pathogen or infection employing three different approaches: (1) case-control malaria cohort from Orissa and Chhattisgarh, the malaria endemic regions of India; (2) a comprehensive assessment in seventy-six ethnically, linguistically and geographically diverse populations inhabited across India; and (3) evaluation of samples from Brazil, Syria and Vietnam and comparison of results with Indian populations.

We observed significant difference in genotype and allele frequency distribution along with haplotype carriage between three groups of malaria case-control study. However, mild and severe groups do not differ among themselves; they differ significantly when compared to asymptomatic control. We also found these regulatory markers are in strong LD (r^2^>0.75) and carriage of the resultant haplotype TTR2 with asymptomatic and CCR3 with malaria cases (OR = 0.552, 95%CI = 0.356–0.854, p = 0.009) (Table S2). Malaria outcome is the result of complex interaction of a large number of factors and the pathogenesis might be regulated by various mechanisms. However, our findings show that the high IL-4 producing haplotype TTR2 protects or increases survival against *Plasmodium falciparum* malaria. It has been reported that the carriers of TT with cerebral malaria had elevated total IgE compared to non-carriers and suggested that the *IL-4* play a regulatory role in the pathogenesis of malaria in Ghanaian children [8]. Further, association between *IL-4* -590T allele and lower prevalence of *Plasmodium falciparum* infection in asymptomatic Fulani population of Mali has been documented [36]. Several findings have shown association of -590T, -34T and intron-3 VNTR polymorphism R2 with high level of serum IL-4 and consequent high level of total IgG, IgE, anti-plasmodium IgG and IgE, and severity of infection in several populations of malaria endemic region across the globe [16], [17], [18], [19], [21], [22], [23], [24], [25], [37].

It has been established that the high level of pro-inflammatory cytokines (TNF-α, IFN-γ) produced during malaria infection leads to severe pathogenesis [38], [39], [40], [41], [42], [43]. These cytokine up-regulates expressions of endothelial adhesion molecules (ICAM-1) in brain and kidney, which facilitates increased sequestration of parasitized RBC within the microvasculature of these organs [38], [39]. This increased sequestration of parasitized RBC leads to cerebral malaria and renal failure. Further, inhibitory effect of TNF-α on erythropoiesis and subsequent severe malarial pathogenesis has been demonstrated in human and mouse model of experimental cerebral malaria (ECM) [41], [42], [43]. In ECM mouse model, treatment with anti-inflammatory cytokine IFN-α and IFN-ß inhibited cerebral malaria and reduced the parasite burden [39], [40]. IFN-ß treatment down regulate pro-inflammatory cytokine TNF-α, IFN-γ, ICAM-1, CXCL9 and CXCR3 [40]. These studies indicate, a check on pro-inflammatory cytokine by anti-inflammatory cytokine can lead to enhanced survival against *Plasmodium falciparum* malaria. This supports our finding that shows high IL-4 producing haplotype, TTR2, provide decreased susceptibility to *Plasmodium* infection.

In contrast to the general accepted view that IL-4 secreting CD4+ T cells are anti-inflammatory mediators and suppress pro-inflammatory response, CD4+ T cells has been found to be crucial to the development of pro-inflammatory CD8+ T cell response against *Plasmodium* sporozoite infected hepatocytes [44], [45], [46]. It has been observed that early development of protective circumsporozoite protein (CSP) specific CD8+ T cell originates in cutaneous lymphoid tissue of infected site and then migrate to other sites including liver [47], [48]. Development of this immunity requires IL-4 mediated cross talk of CD4/CD8 cells [44]. The CSP specific CD8+ T cells, which get primed in presence of IL-4 signals, differentiate into effector memory CD8+ T cells, whereas in absence of IL-4 the response fails to develop further after few days and reduced by more than 90% compared to that in presence of CD4+ T cells. These recent reports further support our findings [44].

In this study, among Indian populations, we found over-representation of R2R2 and R2R3 genotype in ATP while under-representation in caste and *vice-versa*. No significant difference in genotype or allele distribution has been found between caste and nomadic populations. Our Y-chromosomal markers based population study explains that this deviation from general perception is due to recent admixture and gene flow between the caste and nomadic populations (our unpublished data). However, significant difference has been found between all other groups. Every single Indian population maintain its unique genetic architecture; mainly due to endogamy marriage practice over the last thousands of year. This has been well supported by our earlier studies using mtDNA, Y chromosome and autosomal genetic markers [30], [31]. We found that these three markers are in strong LD (r^2^>0.87) with two main haplotypes TTR2 and CCR3. Distribution of protective haplotype TTR2 has been found significantly higher in ATP than tribe and caste while at intermediary in tribe. The ATPs are inhabited in isolated forest and they mainly practice hunter-gatherer lifestyle, hence, they are under constant exposure to helminthes and various other parasites. Therefore, positive selection [20] might be operating on *IL-4* locus of the ATPs compared to caste populations, who practice modern lifestyle and expose to modern medicine.

This is the first study of its kind, where we studied the *IL-4* variations in such a depth in diverse Indian populations. We also observed that a few populations (Bodo, Gadaba, Baiga, Toda, Malai Kuruwar) are significantly departs from HW equilibrium, which might be the result of positive selection or founder effect as their population size are very small and follow very strict endogamy practice.

Apart from its role in controlling malaria and other pathogenic disease, the *IL-4* polymorphisms (-590T, -34T and intron-3 VNTR R2) have been found to be associated with end stage renal disease, multiple sclerosis, autoimmune Grave’s disease, polyarthritis, rheumatoid arthritis, asthma, rhinitis and atopic dermatitis [21], [26], [27], [28], [29]. This Th2 response also mediates inflammatory response to helminth infection [49]. This indicates ATP and tribal populations not only have more survival potential against autoimmune and allergic disease but also against extracellular helminthic infection then the caste populations.

In conclusion, *IL-4* -590T, -34T and intron-3 VNTR R2 allele is associated with enhanced survival against malaria and other extracellular pathogens in Indian populations. However, their role needs to be assessed further for other infectious, inflammatory and autoimmune diseases. This observation may assist in finding individuals at high risk and hence, disease management. These linked marker along with other markers being in LD can cause balance shift of cytokine profile and hence T_H_1 and T_H_2 response in an individual up to an extent, where it can be deleterious also. Thus, a delicate balance of various cytokine is more important than the specific one. Hence, for detailed understanding, other regulators need to be studied among Indian populations. Our study also emphasize the importance of host genetics in resistance/susceptibility to infectious disease.

## Supporting Information

Figure S1 Multiple sequence alignment of *IL4* intron-3 VNTR (70 bp repeat) region of six primates. Mostly two and three copies of repeats has been observed in humans, whereas only a single copy of 70 bp repeat has been observed in other primates (www.ensembl.org).
(TIF)

Figure S2 Pairwise F*st* matrix of 76 studied ethnically, geographically and linguistically different Indian populations.
(TIF)

Figure S3 Comparison of *IL-4* intron-3 VNTR R2/R3 genotype distribution among various caste populations (upper, middle and lower caste).
(TIF)

Figure S4 Frequency of three studied loci (-590 C/T, -34 C/T and intron-3 VNTR) in the present study and various world populations. *Present study
(TIF)

Table S1 Post-hoc analysis of chi-square contingency-table test showing adjusted residual (z-score) value in malaria case control and population groups.
(DOC)

Table S2 Haplotype frequency distribution among various groups of malaria case control and population study.
(DOC)

Table S3 Comparison of IL4 intron-3 VNTR polymorphism R2/R3 between patient and asymptomatic group, stratified by sex
(DOC)
