# Supplementary figures and images for: IL-4 Haplotype -590T, -34T and Intron-3 VNTR R2 Is Associated with Reduced Malaria Risk among Ancestral Indian Tribal Populations
Source: PLoS One. 2012 Oct 24;7(10):e48136. doi: 10.1371/journal.pone.0048136 (PMC3480467; doi:10.1371/journal.pone.0048136)

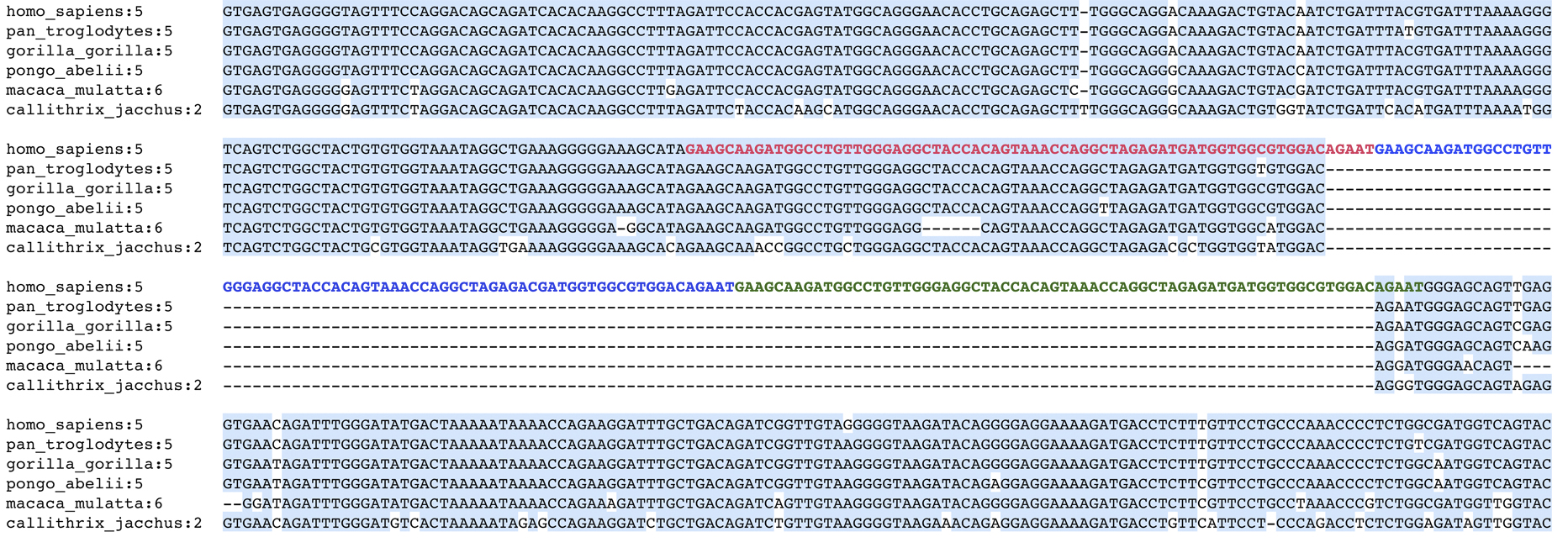

Supplement: Figure S1 — Multiple sequence alignment of IL4 intron-3 VNTR (70 bp repeat) region of six primates. Mostly two and three copies of repeats has been observed in humans, whereas only a single copy of 70 bp repeat has been observed in other primates (www.ensembl.org). (TIF) [file pone.0048136.s001.tif]

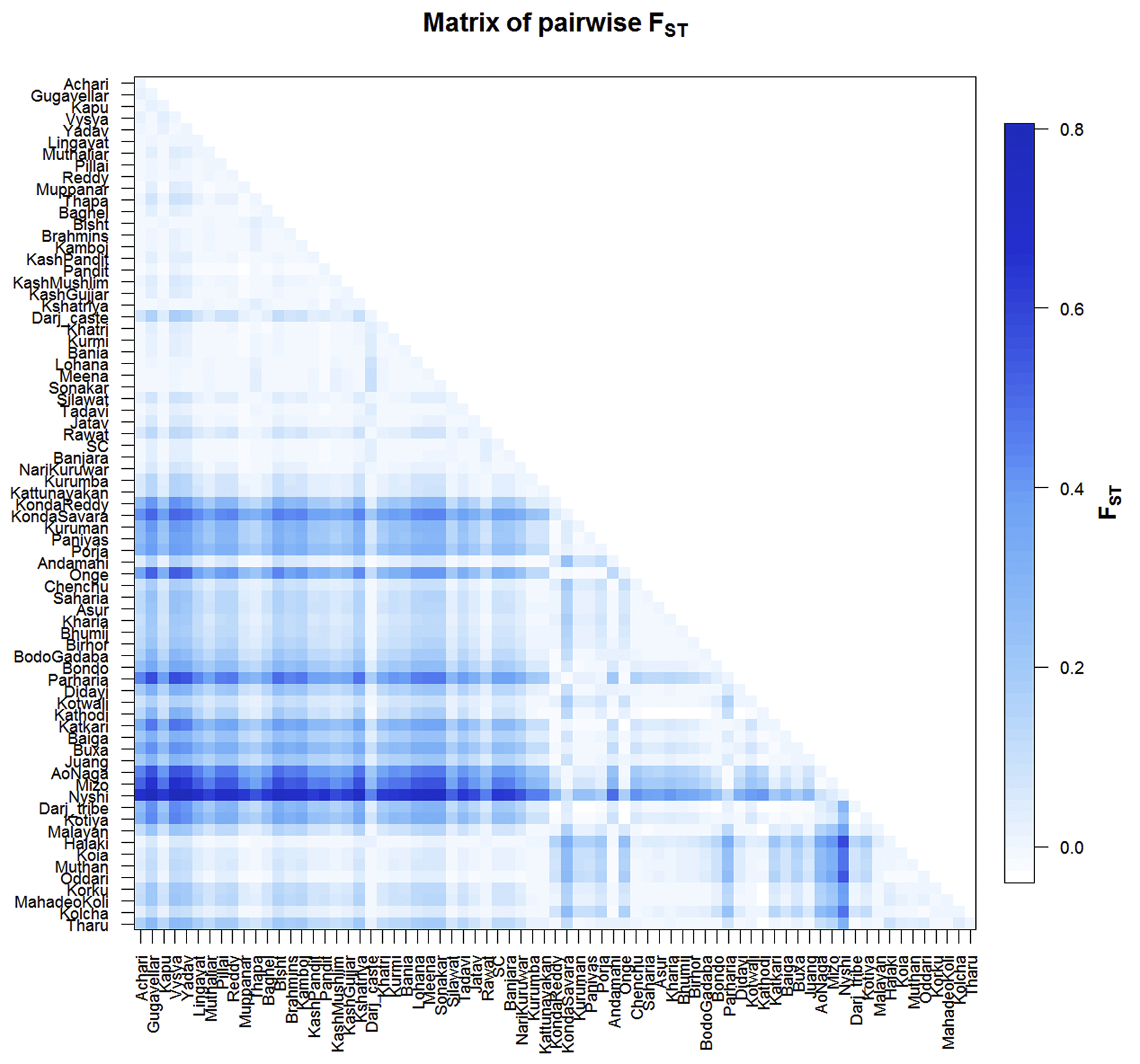

Supplement: Figure S2 — Pairwise Fst matrix of 76 studied ethnically, geographically and linguistically different Indian populations. (TIF) [file pone.0048136.s002.tif]

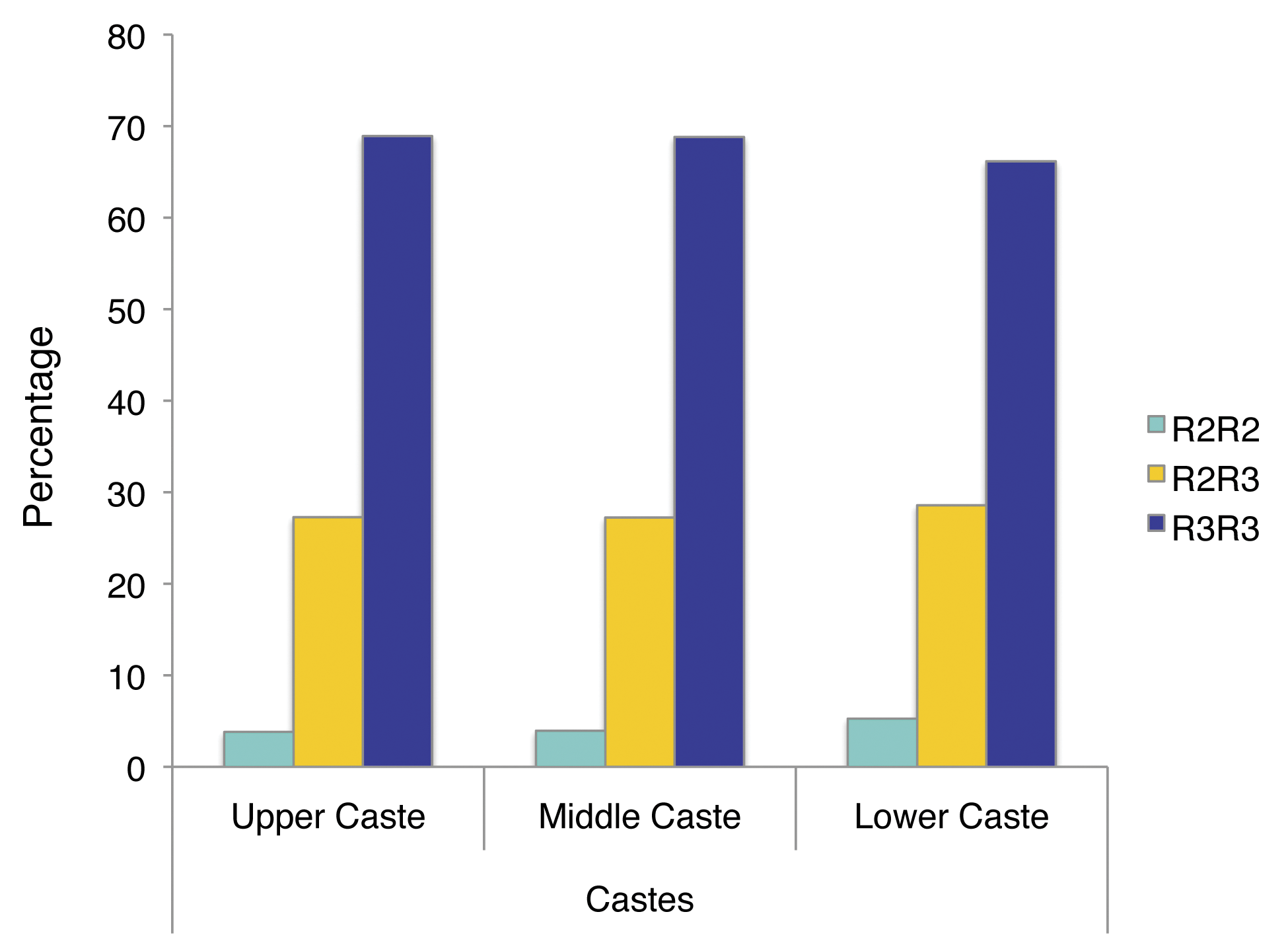

Supplement: Figure S3 — Comparison of IL-4 intron-3 VNTR R2/R3 genotype distribution among various caste populations (upper, middle and lower caste). (TIF) [file pone.0048136.s003.tif]

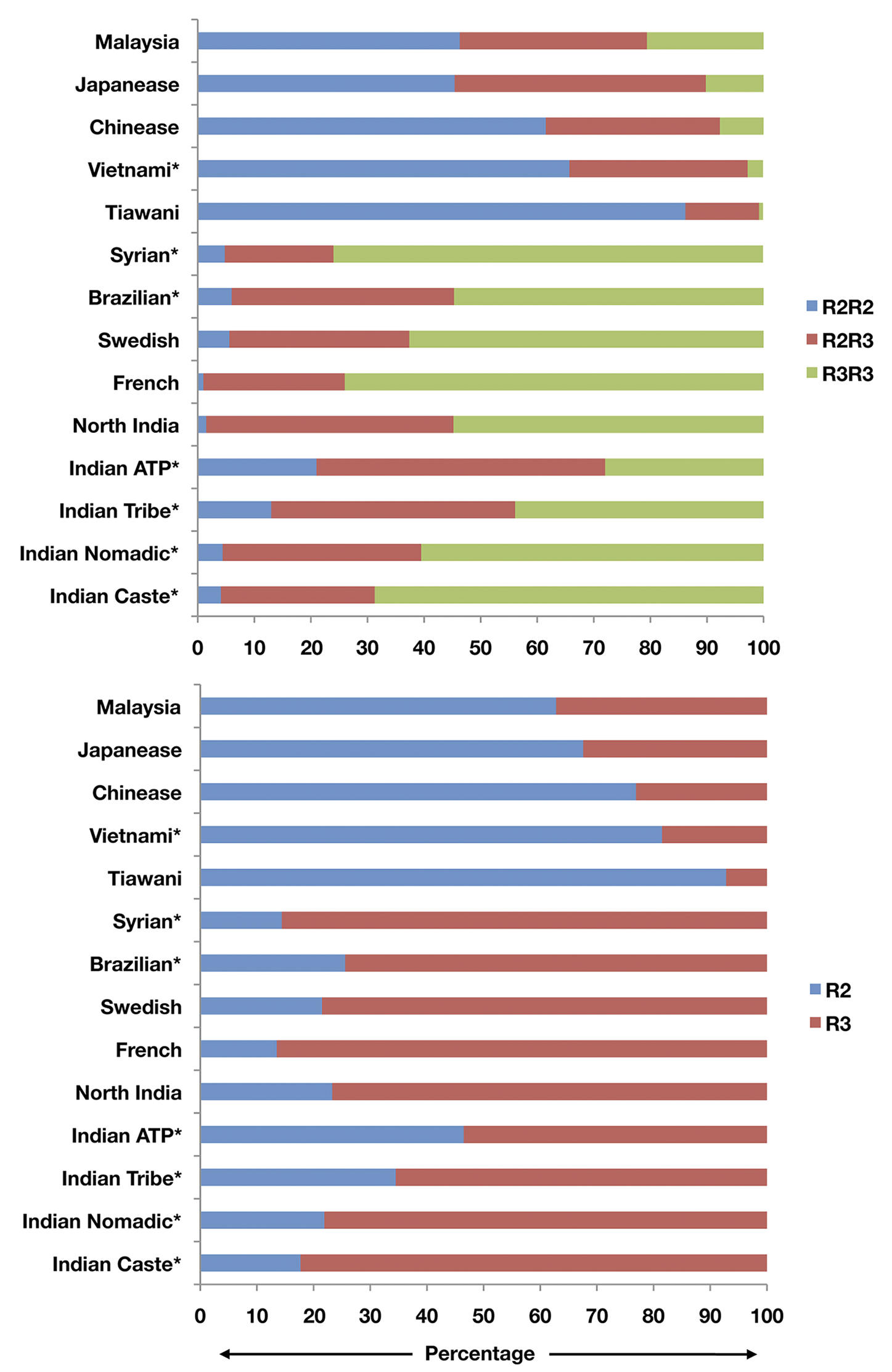

Supplement: Figure S4 — Frequency of three studied loci (-590 C/T, -34 C/T and intron-3 VNTR) in the present study and various world populations. *Present study (TIF) [file pone.0048136.s004.tif]
