# Supplementary material for: IL-4 Haplotype -590T, -34T and Intron-3 VNTR R2 Is Associated with Reduced Malaria Risk among Ancestral Indian Tribal Populations
Source: PLoS One. 2012 Oct 24;7(10):e48136. doi: 10.1371/journal.pone.0048136 (PMC3480467; doi:10.1371/journal.pone.0048136)
Supplement: Table S1 — Post-hoc analysis of chi-square contingency-table test showing adjusted residual (z-score) value in malaria case control and population groups. (DOC) [file pone.0048136.s005.doc]

Supplementary Table 1: Post-hoc analysis of chi-square contingency-table test showing adjusted residual (z-score) value in malaria case control and population groups.

|  | R2R2 | R2R3 | R3R3 |
| --- | --- | --- | --- |
| Malaria case control group | | | |
| Asymptomatic control | 1.1 | 5.5 | -6.1 |
| Mild Malaria | -2.3 | -2.1 | 3.5 |
| Severe Malaria | 0.9 | -3.6 | 3.0 |
| Population groups | | | |
| Caste | -12.3 | -12.2 | 19.6 |
| Nomadic | -2.4 | -0.7 | 2.1 |
| Tribe | 1.3 | 2.6 | -3.4 |
| ATP | 12.9 | 11.1 | -18.9 |

ATP: ancestral tribal population

Two tailed value of z at 0.05 level of significance = 1.98
