# Supplementary material for: IL-4 Haplotype -590T, -34T and Intron-3 VNTR R2 Is Associated with Reduced Malaria Risk among Ancestral Indian Tribal Populations
Source: PLoS One. 2012 Oct 24;7(10):e48136. doi: 10.1371/journal.pone.0048136 (PMC3480467; doi:10.1371/journal.pone.0048136)
Supplement: Table S2 — Haplotype frequency distribution among various groups of malaria case control and population study. (DOC) [file pone.0048136.s006.doc]

Supplementary Table 2: Haplotype frequency distribution among various groups of malaria case control and population study.

|  | Haplotype | | Haplotype Comparison | |
| --- | --- | --- | --- | --- |
|  | CCR3 | TTR2 | χ^2^ | p-value |
| Malaria case-control groups | | | | |
| Asymptomatic Control | 0.632 | 0.348 |  |  |
| Mild Malaria | 0.772 | 0.184 |  |  |
| Severe Malaria | 0.728 | 0.259 |  |  |
| Pooled Cases | 0.746 | 0.228 | 7.2, df = 1 | 0.009 * |
| Population groups | | | | |
| Caste | 0.814 | 0.176 |  |  |
| Nomadic | 0.767 | 0.210 |  |  |
| Tribe | 0.658 | 0.329 |  |  |
| Ancestral Tribe | 0.581 | 0.396 | 182.95, df = 3 | 0.001 |

* Pooled case vs. Asymptomatic, OR = 0.552, 95%CI = 0.356 - 0.854; df: degree of freedom
