# Supplementary material for: IL-4 Haplotype -590T, -34T and Intron-3 VNTR R2 Is Associated with Reduced Malaria Risk among Ancestral Indian Tribal Populations
Source: PLoS One. 2012 Oct 24;7(10):e48136. doi: 10.1371/journal.pone.0048136 (PMC3480467; doi:10.1371/journal.pone.0048136)
Supplement: Table S3 — Comparison of IL4 intron-3 VNTR polymorphism R2/R3 between patient and asymptomatic group, stratified by sex (DOC) [file pone.0048136.s007.doc]

Supplementary Table 3: Comparison of IL4 intron-3 VNTR polymorphism R2/R3 between patient and asymptomatic group, stratified by sex

|  | Genotype | | | |  |  | Allele | | Bootstrap* | | | |
| --- | --- | --- | --- | --- | --- | --- | --- | --- | --- | --- | --- | --- |
|  | No. of samples | R2R2 | R2R3 | R3R3 | χ^2^, df = 2 | p-value | R2 | R3 | OR | Bias | SE | BCa 95% CI |
| Male |  |  |  |  |  |  |  |  |  |  |  |  |
| Asymptomatic | 92 | 10 (10.9) | 53 (57.6) | 29 (31.5) |  |  | 73 (39.7) | 111 (60.3) |  |  |  |  |
| Mild | 67 | 3 (5.3) | 19 (33.3) | 45 (61.4) | 18.9 | < 0.001 | 25 (18.6) | 109 (81.4) | 2.867 | 0.151 | 0.849 | 1.688 – 5.185 |
| Severe | 103 | 12 (11.7) | 28 (27.2) | 63 (61.2) | 19.9 | < 0.001 | 52 (25.2) | 154 (74.8) | 1.948 | 0.062 | 0.456 | 1.269 – 3.081 |
| Female |  |  |  |  |  |  |  |  |  |  |  |  |
| Asymptomatic | 64 | 9 (14.1) | 36 (56.2) | 19 (29.7) |  |  | 54 (42.1) | 74 (57.8) |  |  |  |  |
| Mild | 34 | 1 (2.9) | 12 (35.3) | 21 (61.8) | 10.28 | 0.006 | 14 (20.6) | 54 (79.4) | 2.815 | 0.289 | 0.649 | 1.417 – 6.528 |
| Severe | 70 | 8 (11.4) | 23 (32.8) | 39 (55.7) | 9.57 | 0.008 | 39 (27.9) | 101 (72.1) | 1.890 | 0.072 | 0.536 | 1.116 – 3.214 |

*Based on 10000 random sampling with bias-corrected and accelerated (BCa) and sex as strata variables using SPSS v. 20;

CI: Class Interval; df: degree of freedom;
